# Roles of IL-25 in Type 2 Inflammation and Autoimmune Pathogenesis

**DOI:** 10.3389/fimmu.2021.691559

**Published:** 2021-05-28

**Authors:** Chong Deng, Na Peng, Yuan Tang, Na Yu, Cuicui Wang, Xiaoyan Cai, Lijun Zhang, Dajun Hu, Francesco Ciccia, Liwei Lu

**Affiliations:** ^1^ Department of Pathology and Shenzhen Institute of Research and Innovation, The University of Hong Kong, Hong Kong, China; ^2^ Department of Rheumatology and Nephrology, The Second People’s Hospital, China Three Gorges University, Yichang, China; ^3^ Department of Rheumatology, Guangzhou First People’s Hospital, School of Medicine, South China University of Technology, Guangzhou, China; ^4^ Department of Rheumatology, Shenzhen Hospital, The University of Hong Kong, Shenzhen, China; ^5^ Dipartimento di Medicina di Precisione, Section of Rheumatology, Università degli Studi della Campania L. Vanvitelli, Naples, Italy

**Keywords:** IL-25, IL-25 signal transduction, type 2 inflammation, systemic erythematosus lupus, rheumatoid arthritis

## Abstract

Interleukin-17E (IL-25) is a member of the IL-17 cytokine family that includes IL-17A to IL-17F. IL-17 family cytokines play a key role in host defense responses and inflammatory diseases. Compared with other IL-17 cytokine family members, IL-25 has relatively low sequence similarity to IL-17A and exhibits a distinct function from other IL-17 cytokines. IL-25 binds to its receptor composed of IL-17 receptor A (IL-17RA) and IL-17 receptor B (IL-17RB) for signal transduction. IL-25 has been implicated as a type 2 cytokine and can induce the production of IL-4, IL-5 and IL-13, which in turn inhibits the differentiation of T helper (Th) 17. In addition to its anti-inflammatory properties, IL-25 also exhibits a pro-inflammatory effect in the pathogenesis of Th17-dominated diseases. Here, we review recent advances in the roles of IL-25 in the pathogenesis of inflammation and autoimmune diseases.

## Introduction

The interleukin-17 (IL-17) family belongs to a group of cytokines that play a crucial role in host defense against extracellular pathogens and inflammatory response during autoimmune pathogenesis (1). As the first cytokine identified in IL-17 family, IL-17A, firstly named as cytotoxic T lymphocyte-associated antigen-8 (CTLA-8), encodes a protein with the same homology as the putative protein encoded by the ORF13 gene of herpesvirus Saimiri (2, 3). Based on the sequence of IL-17A, other IL-17 family members are identified, including IL-17B, IL-17C, IL-17D, IL-17E (also known as IL-25) and IL-17F. IL-17 family cytokines exhibit functional activity by covalently binding to form heterodimers or homodimers. IL-17A and IL-17F can form both homodimer and heterodimer, while IL-17B, IL-17C, IL-17D and IL-25 form homodimers to bind receptors (4, 5).

IL-17 family cytokines play an essential role in host defense against pathogens as well as in various diseases including cancers and autoimmune disorders (1, 6). Recent studies have demonstrated that IL-17A and IL-17F act as pro-inflammatory cytokines in the pathogenesis of Sjögren’s syndrome (SS) (7, 8). In addition, IL-17A can sustain plasma cell response and exacerbate the development of systemic lupus erythematosus (SLE) (9). IL-17F has also been shown to drive renal tissue injury in lupus mice, suggesting the pathogenic functions of IL-17A and IL-17F in lupus pathogenesis (10, 11). Moreover, increased levels of IL-17A and IL-17F expression are detected in the inflamed guts of patients with inflammatory bowel disease (IBD) (12, 13). Furthermore, elevated serum IL-17A and increased islet antigen-specific IL-17A-producing CD4^+^ T helper (Th17) cells are detected in patients with type 1 diabetes (T1D) while adoptive transfer of Th17 cells into non-obese diabetic (NOD) mice promotes pancreatic inflammation (14, 15). In multiple sclerosis (MS) patients, IL-17A is found to impair the neural cell function in central nervous system (CNS) and causes tissue destruction (16). Extensive evidence indicates that IL-17A plays a key role in the pathogenesis of psoriasis. IL-17A can induce keratinocytes to produce various chemokines that recruit immune cells and promote the proliferation of endothelial cells, leading to angiogenesis (17). IL-17A is critically involved in the pathogenesis of collagen-induced arthritis (CIA) in mice and rheumatoid arthritis (RA) in patients (18). IL-17A stimulates the synoviocytes to produce vascular endothelial growth factor (VEGF) and induces stromal cells to produce pro-inflammatory cytokines and hematopoietic cytokines (19, 20). As a pro-inflammatory cytokine, IL-17B can recruit neutrophils in immune reactions (21). Elevated levels of IL-17B expression have been found in synovial tissue of CIA mice and RA patients while further blockade of IL-17B with neutralizing antibodies ameliorates disease progression, indicating a pathogenic role of IL-17B in autoimmune diseases (22, 23). Unlike IL-17A, IL-17C is mainly expressed by epithelial cells and can regulate epithelial immune response in an autocrine manner (24, 25). In a dextran sulfate sodium (DSS)-induced colitis mouse model for IBD, IL-17C exhibits a protective role in colitis development (24, 26). However, in mice with imiquimod-induced psoriasis, IL-17C elicits a pathogenic effect and exacerbates psoriatic inflammation, in which intradermal injection of IL-17C triggers leukocyte infiltration and epidermal thickening (24). Thus, IL-17C exerts diverse functions in the development of various autoimmune diseases. Among IL-17 family cytokines, IL-17D is a less studied cytokine, which has been found to induce the expression of pro-inflammatory cytokines including IL-6 and IL-8 in endothelial cells (27). A recent study has identified CD93 as the IL-17D receptor expressed in group 3 innate lymphoid cells (ILC3s) whereas IL-17D exerts anti-inflammatory effects in DSS-induced colitis through inducing IL-22 production by ILC3s (28).

IL-25 was first identified by sequence alignment from human genomic DNA sequence information and considered as a novel proinflammatory cytokine *via* activation through the nuclear factor-κB (NF-κB) (29). Subsequently, IL-25 was defined as a type 2 cytokine produced by Th2 cells, which was capable of inducing IL-4, IL-5 and IL-13 gene expression and further amplifying allergic inflammatory response in the lung and the digestive tract (30). The functions of IL-25 as a “barrier surface” cytokine in epithelial immunology and airway diseases have been recently reviewed (31, 32). Here, we summarize research advances in understanding the roles of IL-25 in inflammation with an emphasis on autoimmune pathogenesis.

## IL-25 and its Signal Transduction

The IL-17 cytokine family binds to its receptors for signal transduction, which include five receptor subunits, IL-17RA, IL-17RB, IL-17RC, IL-17RD and IL-17RE (33). Each IL-17R subunit is a single transmembrane domain-containing protein with several conserved motifs, including extracellular fibronectin III-like motifs, transmembrane regions and cytoplasmic SEF/IL-17R (SEFIR) domains (34). In addition to the SEFIR domain expressed by all IL-17R subunits, IL-17RA also expresses Toll/IL-1R-like loop (TIR-like loop, TILL) domain and C/EBPβ-activation domain (CBAD) (34, 35). IL-17R subunits from both mouse and human range in size from 272 to 866 amino acids and contain full-length forms and smaller alternatively spliced isoforms (36). Since IL-17RA contains most of the cytoplasmic domains, it is the largest member of the IL-17R family and is the key component used at least by IL-17A/IL-17F, IL-17B and IL-25 (37–39). Dimeric IL-17A and IL-17F can bind to receptors consisting of IL-17RA/IL-17RC, IL-17RA/IL-17RD or IL-17RC/IL-17RC (38, 40, 41). In addition, IL-17C uses IL-17RA and IL-17RE to transduce signal (42). Recently, CD93 has been identified as a functional receptor that recognizes IL-17D, but whether CD93 pairs with other receptors to transduce signals from IL-17D requires further investigation (28). Both IL-17B and IL-25 signal through a heterodimeric receptor of IL-17RA and IL-17RB (37, 39). IL-25 shows low affinity for IL-17RA but high affinity for IL-17RB. However, IL-25 can also bind to IL-17RA after it is captured by IL-17RB (43, 44) (**Table 1**).

**Table 1:** IL-17 family cytokines, receptors and functions in autoimmune diseases.

Cytokine	Structure	Receptors	Functions	Ref
IL-17A IL-17F	IL-17A/IL-17A	IL-17RA/IL-17RC	Pathogenic in psoriasis, SLE, SS, T1D, RA, MS and IBD	(7, 9, 12–14, 16, 17, 19, 38, 40, 41)
		IL-17RA/IL-17RD		
	IL-17A/IL-17F	IL-17RA/IL-17RC		
	IL-17F/IL-17F	IL-17RC/IL-17RC		
IL-17B	IL-17B/IL-17B	IL-17RA/IL-17RB	Pathogenic in RA and SLE	(22, 23, 37)
IL-17C	IL-17C/IL-17C	IL-17RA/IL-17RE	Pathogenic in IMQ-induced psoriasis Protective in DSS-induced colitis	(24, 42)
IL-17D	IL-17D/IL-17D	CD93	Protective in DSS-induced colitis	(28)
IL-17E (IL-25)	IL-25/IL-25	IL-17RA/IL-17RB	Pathogenic in psoriasis, SS and type 2 inflammation Protective in IBD, T1D, MS and SLE	(39, 45–50)

SLE, systemic lupus erythematosus; SS, Sjögren’s syndrome; T1D, type 1 diabetes; RA, rheumatoid arthritis; MS, multiple sclerosis; IBD, inflammatory bowel disease; IMQ, imiquimod; DSS, dextran sulfate sodium.

The SEFIR domain is expressed by all IL-17R family members, whereas the TILL domain and CBAD are expressed only by IL-17RA, indicating that IL-17RA might be responsible for more complex signaling process than other IL-17R subunits (34). The SEFIR domain was identified as a conserved segment similar to TIR domain which is known to mediate homotypic interactions (51). Multiple sequence alignments showed that box 1 and box 2 motif in TIR domain are conserved in SEFIR domain, indicating that SEFIR domain-containing protein can interact homotypically with other SEFIR domain-containing proteins (51). A SEFIR domain-containing protein involved in IL-17 cytokine family signaling is activator 1 (Act1), which is an NF-κB activator (52). Act1 can be recruited to IL-17R upon cytokine engagement through SEFIR-SEFIR domain binding (53, 54). Two tumor necrosis factor (TNF) receptor-associated factor-binding (TRAF-binding) sits are shown at the N terminus of Act1, therefore TRAF-containing proteins including TRAF3, TRAF6 and transforming growth factor β-activated kinase 1 (TGFβ-activated kinase 1, TAK1) bind to IL-17R upon engagement (54). TILL domain resembles box 3 motif of TIR domain and are unique in IL-17RA subunit. Mutation of the TILL domain renders mice insufficient response to LPS (34). Another C-terminal domain, CBAD is also unique in IL-17RA subunit, which is required for activation of C/EBPβ and induction of IL-17 target gene expression (34). Signal transduction *via* IL-25 requires heterodimer of IL-17RA and IL-17RB subunits, therefore SEFIR domain, TILL domain and CBAD of IL-17RA as well as SEFIR domain of IL-17RB serve as functional motifs responsible for activation of IL-25 signal (34). Unlike IL-17RA requires Act1 for association, it is reported that IL-17RB can bind TRAF6 directly for the activation of NF-κB (53, 55). However, the activation of mitogen-activated protein kinase (MAPK) including extracellular signal-regulated kinase (ERK), c-Jun N-terminal kinase (JNK) and p38 downstream of IL-25 is independent of TRAF6 (55).

## IL-25 in Type 2 Inflammation and Autoimmune Pathogenesis

### Type 2 Inflammation and Allergic Response

Type 2 inflammation in respiratory system is the hallmark of diseases such as asthma and allergy (56). IL-25, originally identified as a type 2 cytokine produced by Th2 cells, promotes the production of IL-4, IL-5 and IL-13, leading to inflammation in the respiratory tract (30). In addition to Th2 cells as the cellular source, IL-25 may also be derived from group 2 innate lymphoid cells (ILC2s), macrophages, eosinophils, basophils and pulmonary epithelial cells (57). It has been reported that transgenic mice with IL-25 overexpression in pulmonary epithelial cells spontaneously develop asthma-like symptoms, including mucus production and airway infiltration by macrophages and eosinophils (45). Moreover, IL-25 produced by Th2 memory T cells can induce angiogenesis in asthmatic bronchial mucosa (58). Further, blockade of IL-25 significantly reduced antigen-induced infiltration of eosinophils and CD4^+^ T cells in the airways (59). Notably, combined blockade of type 2 cytokine IL-13 and IL-25 was even more effective than blockade alone in reducing infiltration of inflammatory cells in the airways with attenuated airway hyperresponsiveness and tissue remodeling (60). In a mouse model of asthma, natural killer T cells (NKT) with a phenotype of CD4^+^IL-17RB^+^ are able to produce IL-13 and Th2 chemokines in response to IL-25 stimulation and therefore promote airway hyperresponsiveness (61). Recent studies have demonstrated that IL-25 drives the expression of the transcription factor GATA-3 in naïve T cells by potentiating the induction of NFATc1 and JunB (45). Moreover, IL-25 can increase the expression of vascular endothelial growth factor (VEGF) and VEGF receptor *via* activating phosphoinositide 3-kinase/protein kinase B (PI3K/Akt) and ERK/MAPK pathways in endothelial cells (58). As an adaptor protein in the downstream of IL-17 cytokine family, Act1 controls the allergic asthma-like inflammation initiated by IL-25 while depletion of Act1 abolishes the asthma symptom in mice (62). In addition, IL-25 promotes eosinophils to produce monocyte chemoattractant protein-1 (MCP-1), macrophage inflammatory protein-1α (MIP-1α), IL-6 and IL-8 *via* the activation of JNK, p38 MAPK and NF-κB pathways (63). Together, available evidence indicates that IL-25 is critically involved in the development of type 2 inflammation. In a preclinical study, ABM125, an anti-IL-25 monoclonal antibody that neutralize human and mouse IL-25, has shown therapeutic effects in treating virus-induced allergic airway disease (64). Thus, targeting IL-25 or IL-17RB^+^ immune cells may represent a promising strategy for the treatment of allergic inflammation.

### Skin Inflammation

Psoriasis is a typical autoimmune disease of skin inflammation characterized by epidermal hyperplasia, increased angiogenesis and dermal inflammation (65). Although the exact pathogenesis of psoriasis is not clear, it has been suggested that systemic Th1/Th2 imbalance and the involvement of Th17 cells contribute to the initiation and exacerbation of this disease (66). Studies by Senra et al. have demonstrated that IL-25 derived from keratinocytes can directly induce skin inflammation *in vivo* by recruiting neutrophils and activating macrophages (67, 68). IL-25 promotes recruitment of human primary neutrophils by activating human primary macrophages. Moreover, IL-25 stimulates human primary macrophages *via* activation of p38 and NF-κB (67). IL-25 is highly expressed in the skin lesion of patients with psoriasis and in a mouse model of psoriasis. IL-25 is found to promote proliferation of IL-17RB^+^ keratinocytes and exacerbation of psoriasis (46). As the major IL-17RB-expressing cells in psoriasis, keratinocytes can be activated by IL-25 *via* activation of STAT3 transcription factor (46). Notably, blockade of IL-17RA using Brodalumab, a co-receptor for IL-17A, IL-17F and IL-25, has shown high efficacy in the treatment of psoriasis (69). Thus, blockade of IL-25 may represent a promising strategy for targeting skin inflammation.

### Inflammatory Bowel Disease

As a chronic inflammatory disorder of gastrointestinal tract, inflammatory bowel disease (IBD) contains two major idiopathic forms: ulcerative colitis and Crohn’s disease (CD). It has been recognized that dysfunctions of mucosal immune response to commensal bacterial flora, as well as genetic and environmental factors, contribute to the pathogenesis of IBD (70). Using *Campylobacter jejuni* infection and dextran sulfate sodium (DSS) treatment to induce colitis in mice, Jennifer R. O’Hara et al. showed a significant decrease in both IL-25 and IL-17A in mouse colonic homogenates, as well as disrupted Toll-like receptor 9 (TLR9) signaling in apical epithelium, which is responsible for maintaining colonic homeostasis (71). Furthermore, IL-25 production by intestinal epithelial cells inhibits Th17 expansion by suppressing macrophage-derived IL-23 production (72). In addition, IL-25 has been shown to suppress intestinal mucosa CD14^+^ cell-derived IL-12 production (73). In patients with active IBD, IL-25 is significantly decreased in serum and inflamed mucosa. Moreover, *in vitro* studies show that TNF, IFNγ and IL-17A production in IBD CD4^+^ T cells is inhibited by IL-25, which also has an inhibitory function in Th1 and Th17 differentiation (47). Similarly, levels of IL-25 are significantly lower in the intestine of IBD patients than those in normal controls. Consistently, stimulation of normal colonic explants with TNF-α reduced IL-25 synthesis (74). However, treatment with TGF-β1 induces IL-25 production in normal colonic explants (74). Interestingly, IL-25-deficient mice display resistance to DSS-induced colitis while IL-25 upregulates IL-33, IL-6 and TNFα expression in colonic epithelial cells, indicating that IL-25 may contribute to the pathogenesis of IBD (75). Currently, it is unclear how IL-25 exerts dual functions in different cell types or disease stages of IBD. Therefore, further clinical investigations await to validate IL-25 as a therapeutic target for the treatment of patients with IBD.

### Type 1 Diabetes

Type 1 diabetes (T1D) is featured with immune dysregulations including pancreatic β-cell destruction triggered by T cells such as Th1 cells and Th17 cells (76, 77). However, IL-25, as an IL-17 cytokine family member, exhibits an inhibitory effect on the pathogenesis of type 1 diabetes. Studies by Emamaullee et al. have reported that IL-25 administration in non-obese diabetic (NOD) mice with spontaneous T1D onset significantly reduces T cell infiltration in the pancreas and decreases serum autoantibodies with similar effects to anti-IL-17A administration, suggesting a protective role of IL-25 in the pathogenesis of T1D (78). Intriguingly, peripheral blood mononuclear cells (PBMC) from T1D patients display significantly increased IL-25 expression together with enhanced production of IL-17A and IL-6 when compared with healthy donors (79). Thus, further studies are needed to address possible dual functions of IL-25 in mediating inflammatory responses in T1D, which may provide a rationale in therapeutic design of IL-25 blockade for treating T1D patients at different disease stages.

### Rheumatoid Arthritis

Rheumatoid arthritis (RA) is a chronic inflammatory disease characterized by inflammation in synovium, cartilage damage and bone erosion, which further leads to joint destruction. It has been shown that IL-25 is overproduced by RA synovial fibroblasts as a pro-inflammatory cytokine during disease pathogenesis (80). However, IL-25 can also act as a receptor antagonist of IL-17A function, resulting in suppressed Th17 response. Moreover, IL-25 can inhibit IL-22-induced osteoclastogenesis *via* activation of signal transducer and activator of transcription 3 (STAT3) and p38 MAPK pathway (81, 82). Lavocat et al. reported that RA synoviocytes express IL-17RB and also secrete IL-25 while TNFα treatment increases IL-17RB expression (81). IL-25 treatment of fibroblast-like synoviocytes (FLS) from RA patients inhibits p38 phosphorylation whereas IL-25 pretreatment downregulates the phosphorylation of STAT3, p38 and IκB-α triggered by IL-22 stimulation in FLS from RA patients (82). In mice with collagen II-induced arthritis (CIA), IL-25 is significantly increased at the late stage of CIA while IL-17 is increased at the early stage, suggesting that IL-25 and IL-17 may be involved in arthritic progression at different stages of inflammatory responses (83).

### Multiple Sclerosis

Multiple sclerosis (MS) is a chronic autoimmune neurological disease of the central nervous system (CNS), which attacks the myelinated axons and destroys the myelin and axons to varying degrees (84). Th17 cells have been characterized as a major CD4^+^ T cell subpopulation mediating the pathogenesis of MS. Recent studies show that IL-25-deficient (*Il25^-/-^*) mice are highly susceptible to experimental autoimmune encephalomyelitis (EAE), a mouse model for human MS, while neutralization of IL-17A prevents EAE in IL-25-deficient mice, indicating a role of IL-25 in attenuating inflammation by inhibiting Th17 function (48). In addition, IL-25 inhibits T cell-triggered neuronal injury and cell death by reducing expression of lymphocyte function-associated antigen-1 (LFA-1) (85). Moreover, Sonobe Y et al. reported that in TNF-α-induced impairment of blood-brain barrier (BBB) permeability, IL-25 treatment downregulates expression of junction adhesion molecule claudin-5, *via* phosphorylation of protein kinase C epsilon (PKCϵ), suggesting that IL-25 produced by brain capillary endothelial cells can maintain BBB integrity (86). Together, available evidence indicates a protective role of IL-25 in the development of MS.

### Systemic Lupus Erythematosus

Systemic Lupus Erythematosus (SLE) is a systemic autoimmune disease involving multiple organs including kidney and brain, characterized by anti-nuclear autoantibody (ANA) and immune complex deposition in kidney, which further causes immune-complex glomerulonephritis (87, 88). Several studies show that IL-25, together with other Th2-related cytokines, is significantly increased in SLE patients, especially in those with lupus nephritis, contributing to the pathogenesis of SLE (89, 90). Although IL-25 is upregulated in SLE patients, IL-25 can ameliorate lupus pathogenesis in mice by inhibiting inflammatory cytokines (49). We have recently identified a critical role of IL-17 in maintaining plasma cell survival and autoantibody production in both SLE patients and murine lupus (9). Currently, it is unclear whether IL-25 modulates the multiple functions of various B cell subsets in autoimmune pathogenesis (91). Thus, further investigation is needed to determine whether IL-25 plays a pro-inflammatory or anti-inflammatory role during the development of SLE.

### Sjögren’s Syndrome

Primary Sjögren’s syndrome (pSS) is characterized as a systemic autoimmune disease with progressive inflammation of salivary glands (SG) and lacrimal glands, which leads to dry mouth and dry eyes (92). Our previous studies have demonstrated that Th17 cells are important in initiating the pathogenesis of SS, indicating a key role for IL-17A in SS (7). Recently, we observed significantly increased expression of IL-25 in SG and peripheral blood from pSS patients compared with healthy controls (50). In culture, IL-25 significantly increases the number of IL-17RB^+^ inflammatory ILC2s (iILC2s) from SG and peripheral blood (50). Furthermore, blockade of IL-25 using a neutralizing antibody markedly improves saliva flow rate and ameliorates SG tissue damage in mice with experimental SS (ESS), accompanied with decreased ILC2 infiltration in SG of ESS mice. In SGs of pSS patients, significant upregulation of TRAF6 in CD3^+^ T cells and ILC2s suggests that IL-25 signal is functional *via* coordinating activation of ERK1/2 and relative transcription factors (50). Recent studies show that ILC2 provokes inflammation in airways causing persistent asthma symptoms, which can be activated by IL-25. Therefore, available studies have indicated that IL-25 plays a pathogenic role during the development of SS (50, 93).

## Conclusion and Perspective

**Figure 1:**
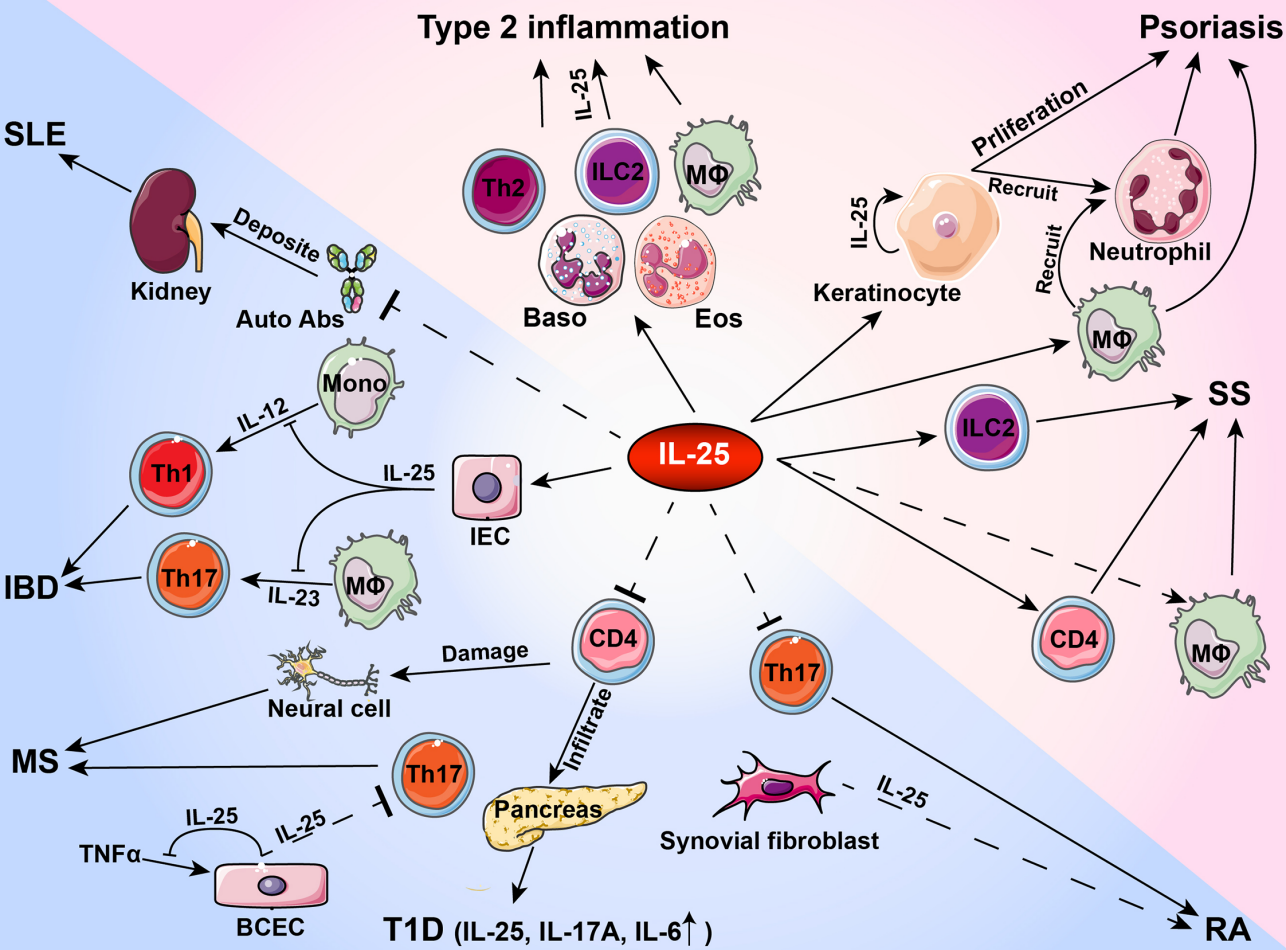
Roles of IL-25 in type 2 inflammation and autoimmune pathogenesis. During the development of autoimmune diseases, IL-25 plays a pro-inflammatory or anti-inflammatory role in activating or inhibiting immune cells and tissue cells. Auto Abs, autoantibodies; Baso, basophil; BCEC, brain capillary epithelial cell; Eos, eosinophil; IBD, inflammatory bowel disease; IEC, intestinal endothelial cell; ILC2, group 2 innate lymphoid cells; SS, Sjögren’s syndrome; MΦ, macrophage; Mono, monocyte; MS, multiple sclerosis; RA, rheumatoid arthritis; SLE, systemic lupus erythematosus; T1D, type 1 diabetes.

As a member of IL-17 cytokine family, IL-25 acts primarily as a type 2 cytokine and is functionally distinct from other IL-17 cytokines. In inflammation and autoimmune pathogenesis, IL-25 binds to receptor subunit IL-17RB expression in immune cells and tissue cells whereas IL-25 levels increase in peripheral blood and inflammatory microenvironment. Current studies suggest that IL-25 has a dual role in regulating immune responses during the development of autoimmune diseases. As a pro-inflammatory cytokine, IL-25 exacerbates allergic inflammation by promoting the production of type 2 cytokines including IL-4, IL-5 and IL-13 by Th2 cells. Moreover, IL-25 activates innate immune cells and induces proliferation, production of other pro-inflammatory cytokines and recruitment of immune cells in psoriasis and SS. In contrast, IL-25 exerts anti-inflammatory effects by inhibiting Th1 or Th17 differentiation *via* production of Th2 cytokines in IBD, T1D, RA, MS and SLE (**Table 2** and **Figure 1**). Given that IL-25 exerts dual functions in various autoimmune diseases, further investigations are needed to determine the exact roles played by IL-25 at different stages of inflammatory responses and autoimmune diseases. Increasing evidence indicates the functional diversities of both B cells and T cells in autoimmune pathogenesis. Future studies on the roles of IL-25 in regulating immune responses may contribute to the design of new therapeutic interventions by targeting IL-25 for the treatment of inflammatory disorders.

**Table 2:** IL-25 in inflammatory and autoimmune disorders.

Disease	Effect	Change	Signaling Pathways	Ref
Allergies	Pro-inflammatory	Increase	NFATc1/JunB-GATA3; PI3K/AKT; ERK; JNK; p38; NF-κB	(45, 58, 63)
Psoriasis	Pro-inflammatory	Increase	JAK/STAT3; p38; NF-κB	(46, 67)
SS	Pro-inflammatory	Increase	ERK	(50)
IBD	Pro-/Anti-inflammatory	Decrease	N/A	(47)
T1D	Anti-inflammatory	Increase	PI3K/AKT; p38; ERK	(94)
MS	Anti-inflammatory	Increase	PKC-claudin-5	(86)
RA	Pro-/Anti-inflammatory	Increase	JAK/STAT3; p38	(82)
SLE	Anti-inflammatory	Increase	N/A	(49)

SS, Sjögren’s syndrome; IBD, inflammatory bowel disease; T1D, type 1 diabetes; MS, multiple sclerosis; RA, rheumatoid arthritis; SLE, systemic lupus erythematosus.

## Author Contributions 

All authors listed have made a substantial, direct, and intellectual contribution to the work, and approved it for publication.

## Funding

This work was supported by the National Natural Science Foundation of China (NSFC) (82071817), Hong Kong Research Grants Council General Research Fund (17113319) and Theme-Based Research Scheme (T12-703/19R), Health Research Fund from Yichang Science and Technology Bureau (A20-2-035) and HKU Seed Funding for Strategic Interdisciplinary Research Scheme.

## Conflict of Interest

The authors declare that the research was conducted in the absence of any commercial or financial relationships that could be construed as a potential conflict of interest.

## References

[1] Dong C. Cytokine Regulation and Function in T Cells. Annu Rev Immunol (2021) 39:51–76. 10.1146/annurev-immunol-061020-053702 33428453

[2] Yao Z Fanslow WC Seldin MF Rousseau AM Painter SL Comeau MR. Herpesvirus Saimiri Encodes a New Cytokine, IL-17, Which Binds to a Novel Cytokine Receptor. Immunity (1995) 3(6):811–21. 10.1016/1074-7613(95)90070-5 8777726

[3] Rouvier E Luciani MF Mattei MG Denizot F Golstein P. Ctla-8, Cloned From an Activated T Cell, Bearing AU-Rich Messenger RNA Instability Sequences, and Homologous to a Herpesvirus Saimiri Gene. J Immunol (1993) 150(12):5445–56. 8390535

[4] Truchetet ME Mossalayi MD Boniface K. IL-17 in the Rheumatologist’s Line of Sight. BioMed Res Int (2013) 2013:295132. 10.1155/2013/295132 23984335 PMC3741932

[5] Wright JF Guo Y Quazi A Luxenberg DP Bennett F Ross JF. Identification of an Interleukin 17f/17a Heterodimer in Activated Human Cd4+ T Cells. J Biol Chem (2007) 282(18):13447–55. 10.1074/jbc.M700499200 17355969

[6] Wang X Lin X Zheng Z Lu B Wang J Tan AH. Host-Derived Lipids Orchestrate Pulmonary Gammadelta T Cell Response to Provide Early Protection Against Influenza Virus Infection. Nat Commun (2021) 12(1):1914. 10.1038/s41467-021-22242-9 33772013 PMC7997921

[7] Lin X Rui K Deng J Tian J Wang X Wang S. Th17 Cells Play a Critical Role in the Development of Experimental Sjogren’s Syndrome. Ann Rheum Dis (2015) 74(6):1302–10. 10.1136/annrheumdis-2013-204584 24573745

[8] Gan Y Zhao X He J Liu X Li Y Sun X. Increased Interleukin-17F Is Associated With Elevated Autoantibody Levels and More Clinically Relevant Than Interleukin-17A in Primary Sjogren’s Syndrome. J Immunol Res (2017) 2017:4768408. 10.1155/2017/4768408 28210632 PMC5292172

[9] Ma K Du W Xiao F Han M Huang E Peng N. IL-17 Sustains the Plasma Cell Response Via P38-Mediated Bcl-Xl RNA Stability in Lupus Pathogenesis. Cell Mol Immunol (2020). 10.1038/s41423-020-00540-4 PMC8245411 32917979

[10] Riedel JH Paust HJ Krohn S Turner JE Kluger MA Steinmetz OM. Il-17f Promotes Tissue Injury in Autoimmune Kidney Diseases. J Am Soc Nephrol (2016) 27(12):3666–77. 10.1681/ASN.2015101077 PMC5118482 27030744

[11] Li D Guo B Wu H Tan L Chang C Lu Q. Interleukin-17 in Systemic Lupus Erythematosus: A Comprehensive Review. Autoimmunity (2015) 48(6):353–61. 10.3109/08916934.2015.1037441 25894789

[12] Galvez J. Role of Th17 Cells in the Pathogenesis of Human Ibd. ISRN Inflammation (2014) 2014:928461. 10.1155/2014/928461 25101191 PMC4005031

[13] Seiderer J Elben I Diegelmann J Glas J Stallhofer J Tillack C. Role of the Novel Th17 Cytokine IL-17F in Inflammatory Bowel Disease (Ibd): Upregulated Colonic IL-17F Expression in Active Crohn’s Disease and Analysis of the IL17F P.His161Arg Polymorphism in IBD. Inflammation Bowel Dis (2008) 14(4):437–45. 10.1002/ibd.20339 18088064

[14] Baharlou R Ahmadi-Vasmehjani A Davami MH Faraji F Atashzar MR Karimipour F. Elevated Levels of T-Helper 17-Associated Cytokines in Diabetes Type I Patients: Indicators for Following the Course of Disease. Immunol Invest (2016) 45(7):641–51. 10.1080/08820139.2016.1197243 27611173

[15] Martin-Orozco N Chung Y Chang SH Wang YH Dong C. Th17 Cells Promote Pancreatic Inflammation But Only Induce Diabetes Efficiently in Lymphopenic Hosts After Conversion Into Th1 Cells. Eur J Immunol (2009) 39(1):216–24. 10.1002/eji.200838475 PMC2755057 19130584

[16] Kolbinger F Huppertz C Mir A Padova FD. IL-17A and Multiple Sclerosis: Signaling Pathways, Producing Cells and Target Cells in the Central Nervous System. Curr Drug Targets (2016) 17(16):1882–93. 10.2174/1389450117666160307144027 26953244

[17] AbuHilal M Walsh S Shear N. The Role of IL-17 in the Pathogenesis of Psoriasis and Update on IL-17 Inhibitors for the Treatment of Plaque Psoriasis. J Cutan Med Surg (2016) 20(6):509–16. 10.1177/1203475416651605 27207350

[18] Lai Kwan Lam Q King Hung Ko O Zheng BJ Lu L. Local BAFF Gene Silencing Suppresses Th17-Cell Generation and Ameliorates Autoimmune Arthritis. Proc Natl Acad Sci USA (2008) 105(39):14993–8. 10.1073/pnas.0806044105 PMC2567481 18820032

[19] Chabaud M Durand JM Buchs N Fossiez F Page G Frappart L. Human Interleukin-17: A T Cell-Derived Proinflammatory Cytokine Produced by the Rheumatoid Synovium. Arthritis Rheum (1999) 42(5):963–70. 10.1002/1529-0131(199905)42:5<963::AID-ANR15>3.0.CO;2-E 10323452

[20] Fossiez F Djossou O Chomarat P Flores-Romo L Ait-Yahia S Maat C. T Cell Interleukin-17 Induces Stromal Cells to Produce Proinflammatory and Hematopoietic Cytokines. J Exp Med (1996) 183(6):2593–603. 10.1084/jem.183.6.2593 PMC2192621 8676080

[21] Shi Y Ullrich SJ Zhang J Connolly K Grzegorzewski KJ Barber MC. A Novel Cytokine Receptor-Ligand Pair. Identification, Molecular Characterization, and In Vivo Immunomodulatory Activity. J Biol Chem (2000) 275(25):19167–76. 10.1074/jbc.M910228199 10749887

[22] Kouri VP Olkkonen J Ainola M Li TF Bjorkman L Konttinen YT. Neutrophils Produce Interleukin-17B in Rheumatoid Synovial Tissue. Rheumatol (Oxford) (2014) 53(1):39–47. 10.1093/rheumatology/ket309 24056520

[23] Robak E Kulczycka-Siennicka L Gerlicz Z Kierstan M Korycka-Wolowiec A Sysa-Jedrzejowska A. Correlations Between Concentrations of Interleukin (IL)-17A, IL-17B and IL-17F, and Endothelial Cells and Proangiogenic Cytokines in Systemic Lupus Erythematosus Patients. Eur Cytokine Netw (2013) 24(1):60–8. 10.1684/ecn.2013.0330 23661335

[24] Ramirez-Carrozzi V Sambandam A Luis E Lin Z Jeet S Lesch J. IL-17C Regulates the Innate Immune Function of Epithelial Cells in an Autocrine Manner. Nat Immunol (2011) 12(12):1159–66. 10.1038/ni.2156 21993848

[25] Li H Chen J Huang A Stinson J Heldens S Foster J. Cloning and Characterization of IL-17B and IL-17C, Two New Members of the IL-17 Cytokine Family. Proc Natl Acad Sci USA (2000) 97(2):773–8. 10.1073/pnas.97.2.773 PMC15406 10639155

[26] Reynolds JM Martinez GJ Nallaparaju KC Chang SH Wang YH Dong C. Cutting Edge: Regulation of Intestinal Inflammation and Barrier Function by IL-17C. J Immunol (2012) 189(9):4226–30. 10.4049/jimmunol.1103014 PMC3478486 23024280

[27] Starnes T Broxmeyer HE Robertson MJ Hromas R. Cutting Edge: IL-17D, a Novel Member of the IL-17 Family, Stimulates Cytokine Production and Inhibits Hemopoiesis. J Immunol (2002) 169(2):642–6. 10.4049/jimmunol.169.2.642 12097364

[28] Huang J Lee HY Zhao X Han J Su Y Sun Q. Interleukin-17D Regulates Group 3 Innate Lymphoid Cell Function Through Its Receptor CD93. Immunity (2021) 54(4):673–86.e4. 10.1016/j.immuni.2021.03.018 33852831

[29] Lee J Ho WH Maruoka M Corpuz RT Baldwin DT Foster JS. IL-17E, a Novel Proinflammatory Ligand for the IL-17 Receptor Homolog IL-17Rh1. J Biol Chem (2001) 276(2):1660–4. 10.1074/jbc.M008289200 11058597

[30] Fort MM Cheung J Yen D Li J Zurawski SM Lo S. IL-25 Induces IL-4, IL-5, and IL-13 and Th2-Associated Pathologies In Vivo. Immunity (2001) 15(6):985–95. 10.1016/S1074-7613(01)00243-6 11754819

[31] Borowczyk J Shutova M Brembilla NC Boehncke WH. IL-25 (IL-17E) in Epithelial Immunology and Pathophysiology. J Allergy Clin Immunol (2021) S0091-6749(20):32417–9. 10.1016/j.jaci.2020.12.628 33485651

[32] Hong H Liao S Chen F Yang Q Wang DY. Role of IL-25, IL-33, and TSLP in Triggering United Airway Diseases Toward Type 2 Inflammation. Allergy (2020) 75(11):2794–804. 10.1111/all.14526 32737888

[33] Gu C Wu L Li X. IL-17 Family: Cytokines, Receptors and Signaling. Cytokine (2013) 64(2):477–85. 10.1016/j.cyto.2013.07.022 PMC3867811 24011563

[34] Maitra A Shen F Hanel W Mossman K Tocker J Swart D. Distinct Functional Motifs Within the IL-17 Receptor Regulate Signal Transduction and Target Gene Expression. Proc Natl Acad Sci USA (2007) 104(18):7506–11. 10.1073/pnas.0611589104 PMC1863505 17456598

[35] Shen F Li N Gade P Kalvakolanu DV Weibley T Doble B. IL-17 Receptor Signaling Inhibits C/Ebpbeta by Sequential Phosphorylation of the Regulatory 2 Domain. Sci Signal (2009) 2(59):ra8. 10.1126/scisignal.2000066 19244213 PMC2754870

[36] Gaffen SL. Structure and Signalling in the IL-17 Receptor Family. Nat Rev Immunol (2009) 9(8):556–67. 10.1038/nri2586 PMC2821718 19575028

[37] Ramirez-Carrozzi V Ota N Sambandam A Wong K Hackney J Martinez-Martin N. Cutting Edge: IL-17b Uses IL-17RA and IL-17RB to Induce Type 2 Inflammation From Human Lymphocytes. J Immunol (2019) 202(7):1935–41. 10.4049/jimmunol.1800696 30770417

[38] Wright JF Bennett F Li B Brooks J Luxenberg DP Whitters MJ. The Human IL-17f/IL-17a Heterodimeric Cytokine Signals Through the IL-17RA/IL-17RC Receptor Complex. J Immunol (2008) 181(4):2799–805. 10.4049/jimmunol.181.4.2799 18684971

[39] Rickel EA Siegel LA Yoon BR Rottman JB Kugler DG Swart DA. Identification of Functional Roles for Both IL-17RB and IL-17RA in Mediating IL-25-Induced Activities. J Immunol (2008) 181(6):4299–310. 10.4049/jimmunol.181.6.4299 18768888

[40] Goepfert A Lehmann S Blank J Kolbinger F Rondeau JM. Structural Analysis Reveals That the Cytokine IL-17f Forms a Homodimeric Complex With Receptor IL-17RC to Drive IL-17ra-Independent Signaling. Immunity (2020) 52(3):499–512.e5. 10.1016/j.immuni.2020.02.004 32187518

[41] Su Y Huang J Zhao X Lu H Wang W Yang XO. Interleukin-17 Receptor D Constitutes an Alternative Receptor for Interleukin-17A Important in Psoriasis-Like Skin Inflammation. Sci Immunol (2019) 4(36):eaau9657. 10.1126/sciimmunol.aau9657 31175175

[42] Chang SH Reynolds JM Pappu BP Chen G Martinez GJ Dong C. Interleukin-17C Promotes Th17 Cell Responses and Autoimmune Disease Via Interleukin-17 Receptor E. Immunity (2011) 35(4):611–21. 10.1016/j.immuni.2011.09.010 PMC5800502 21982598

[43] Hymowitz SG Filvaroff EH Yin JP Lee J Cai L Risser P. IL-17s Adopt a Cystine Knot Fold: Structure and Activity of a Novel Cytokine, IL-17F, and Implications for Receptor Binding. EMBO J (2001) 20(19):5332–41. 10.1093/emboj/20.19.5332 PMC125646 11574464

[44] Ely LK Fischer S Garcia KC. Structural Basis of Receptor Sharing by Interleukin 17 Cytokines. Nat Immunol (2009) 10(12):1245–51. 10.1038/ni.1813 PMC2783927 19838198

[45] Angkasekwinai P Park H Wang YH Wang YH Chang SH Corry DB. Interleukin 25 Promotes the Initiation of Proallergic Type 2 Responses. J Exp Med (2007) 204(7):1509–17. 10.1084/jem.20061675 PMC2118650 17562814

[46] Xu M Lu H Lee YH Wu Y Liu K Shi Y. An Interleukin-25-Mediated Autoregulatory Circuit in Keratinocytes Plays a Pivotal Role in Psoriatic Skin Inflammation. Immunity (2018) 48(4):787–98.e4. 10.1016/j.immuni.2018.03.019 29653697

[47] Su J Chen T Ji XY Liu C Yadav PK Wu R. IL-25 Downregulates Th1/Th17 Immune Response in an IL-10-Dependent Manner in Inflammatory Bowel Disease. Inflammation Bowel Dis (2013) 19(4):720–8. 10.1097/MIB.0b013e3182802a76 23429464

[48] Kleinschek MA Owyang AM Joyce-Shaikh B Langrish CL Chen Y Gorman DM. IL-25 Regulates Th17 Function in Autoimmune Inflammation. J Exp Med (2007) 204(1):161–70. 10.1084/jem.20061738 PMC2118427 17200411

[49] Li Y Wang R Liu S Liu J Pan W Li F. Interleukin-25 Is Upregulated in Patients With Systemic Lupus Erythematosus and Ameliorates Murine Lupus by Inhibiting Inflammatory Cytokine Production. Int Immunopharmacol (2019) 74:105680. 10.1016/j.intimp.2019.105680 31200339

[50] Guggino G Lin X Rizzo A Xiao F Saieva L Raimondo S. Interleukin-25 Axis Is Involved in the Pathogenesis of Human Primary and Experimental Murine Sjogren’s Syndrome. Arthritis Rheumatol (2018) 70(8):1265–75. 10.1002/art.40500 29569854

[51] Novatchkova M Leibbrandt A Werzowa J Neubuser A Eisenhaber F. The STIR-Domain Superfamily in Signal Transduction, Development and Immunity. Trends Biochem Sci (2003) 28(5):226–9. 10.1016/S0968-0004(03)00067-7 12765832

[52] Lv F Song LJ Wang XH Qiu F Li XF. The Role of Act1, a NF-kappaB-activating Protein, in IL-6 and IL-8 Levels Induced by IL-17 Stimulation in SW982 Cells. Pharm Biol (2013) 51(11):1444–50. 10.3109/13880209.2013.798668 23862741

[53] May MJ. IL-17R Signaling: New Players Get in on the Act1. Nat Immunol (2011) 12(9):813–5. 10.1038/ni.2093 21852777

[54] Qian Y Liu C Hartupee J Altuntas CZ Gulen MF Jane-Wit D. The Adaptor Act1 Is Required for Interleukin 17-Dependent Signaling Associated With Autoimmune and Inflammatory Disease. Nat Immunol (2007) 8(3):247–56. 10.1038/ni1439 17277779

[55] Maezawa Y Nakajima H Suzuki K Tamachi T Ikeda K Inoue J. Involvement of TNF Receptor-Associated Factor 6 in IL-25 Receptor Signaling. J Immunol (2006) 176(2):1013–8. 10.4049/jimmunol.176.2.1013 16393988

[56] Patel NN Kohanski MA Maina IW Workman AD Herbert DR Cohen NA. Sentinels At the Wall: Epithelial-Derived Cytokines Serve as Triggers of Upper Airway Type 2 Inflammation. Int Forum Allergy Rhinol (2019) 9(1):93–9. 10.1002/alr.22206 PMC6318004 30260580

[57] Yao X Sun Y Wang W Sun Y. Interleukin (IL)-25: Pleiotropic Roles in Asthma. Respirology (2016) 21(4):638–47. 10.1111/resp.12707 26699081

[58] Corrigan CJ Wang W Meng Q Fang C Wu H Reay V. T-Helper Cell Type 2 (Th2) Memory T Cell-Potentiating Cytokine IL-25 Has the Potential to Promote Angiogenesis in Asthma. Proc Natl Acad Sci USA (2011) 108(4):1579–84. 10.1073/pnas.1014241108 PMC3029682 21205894

[59] Tamachi T Maezawa Y Ikeda K Kagami S Hatano M Seto Y. IL-25 Enhances Allergic Airway Inflammation by Amplifying a TH2 Cell-Dependent Pathway in Mice. J Allergy Clin Immunol (2006) 118(3):606–14. 10.1016/j.jaci.2006.04.051 16950278

[60] Zhang FQ Han XP Zhang F Ma X Xiang D Yang XM. Therapeutic Efficacy of a Co-Blockade of IL-13 and IL-25 on Airway Inflammation and Remodeling in a Mouse Model of Asthma. Int Immunopharmacol (2017) 46:133–40. 10.1016/j.intimp.2017.03.005 28282577

[61] Terashima A Watarai H Inoue S Sekine E Nakagawa R Hase K. A Novel Subset of Mouse Nkt Cells Bearing the IL-17 Receptor B Responds to IL-25 and Contributes to Airway Hyperreactivity. J Exp Med (2008) 205(12):2727–33. 10.1084/jem.20080698 PMC2585837 19015310

[62] Claudio E Sonder SU Saret S Carvalho G Ramalingam TR Wynn TA. The Adaptor Protein CIKS/Act1 Is Essential for IL-25-mediated Allergic Airway Inflammation. J Immunol (2009) 182(3):1617–30. 10.4049/jimmunol.182.3.1617 PMC2630122 19155511

[63] Wong CK Cheung PF Ip WK Lam CW. Interleukin-25-Induced Chemokines and Interleukin-6 Release From Eosinophils Is Mediated by P38 Mitogen-Activated Protein Kinase, C-Jun N-terminal Kinase, and Nuclear Factor-Kappab. Am J Respir Cell Mol Biol (2005) 33(2):186–94. 10.1165/rcmb.2005-0034OC 15860795

[64] Bartlett N GJ Williams T Vincent T Jackson C Alton K Shimkets R. Abm125 Anti-IL-25 Antibody Pre-Clinical Development for Viral Asthma Exacerbations Identifies IL-25 Mediated Regulation of Type-2-and Anti-Viral Immunity. In: *C31 Mechanistic Insights Into Lung Infection, vol. 197.* Am Thoracic Soc (2018). p. A7759–A7759.

[65] Monteleone G Pallone F MacDonald TT Chimenti S Costanzo A. Psoriasis: From Pathogenesis to Novel Therapeutic Approaches. Clin Sci (Lond) (2011) 120(1):1–11. 10.1042/CS20100163 20846119

[66] Jain S Kaur IR Das S Bhattacharya SN Singh A. T Helper 1 to T Helper 2 Shift in Cytokine Expression: An Autoregulatory Process in Superantigen-Associated Psoriasis Progression? J Med Microbiol (2009) 58(Pt 2):180–4. 10.1099/jmm.0.003939-0 19141734

[67] Senra L Mylonas A Kavanagh RD Fallon PG Conrad C Borowczyk-Michalowska J. IL-17E (IL-25) Enhances Innate Immune Responses During Skin Inflammation. J Invest Dermatol (2019) 139(8):1732–42.e17. 10.1016/j.jid.2019.01.021 30738055

[68] Senra L Stalder R Alvarez Martinez D Chizzolini C Boehncke WH Brembilla NC. Keratinocyte-Derived IL-17E Contributes to Inflammation in Psoriasis. J Invest Dermatol (2016) 136(10):1970–80. 10.1016/j.jid.2016.06.009 27329229

[69] Galluzzo M D’Adamio S Bianchi L Talamonti M. Brodalumab for the Treatment of Psoriasis. Expert Rev Clin Immunol (2016) 12(12):1255–71. 10.1080/1744666X.2016.1246957 27718760

[70] Xavier RJ Podolsky DK. Unravelling the Pathogenesis of Inflammatory Bowel Disease. Nature (2007) 448(7152):427–34. 10.1038/nature06005 17653185

[71] O’Hara JR Feener TD Fischer CD Buret AG. Campylobacter Jejuni Disrupts Protective Toll-Like Receptor 9 Signaling in Colonic Epithelial Cells and Increases the Severity of Dextran Sulfate Sodium-Induced Colitis in Mice. Infect Immun (2012) 80(4):1563–71. 10.1128/IAI.06066-11 PMC3318425 22311925

[72] Zaph C Du Y Saenz SA Nair MG Perrigoue JG Taylor BC. Commensal-Dependent Expression of IL-25 Regulates the IL-23-IL-17 Axis in the Intestine. J Exp Med (2008) 205(10):2191–8. 10.1084/jem.20080720 PMC2556798 18762568

[73] Caruso R Sarra M Stolfi C Rizzo A Fina D Fantini MC. Interleukin-25 Inhibits Interleukin-12 Production and Th1 Cell-Driven Inflammation in the Gut. Gastroenterology (2009) 136(7):2270–9. 10.1053/j.gastro.2009.02.049 19505427

[74] Fina D Franze E Rovedatti L Corazza GR Biancone L Sileri PP. Interleukin-25 Production Is Differently Regulated by TNF-Alpha and TGF-Beta1 in the Human Gut. Mucosal Immunol (2011) 4(2):239–44. 10.1038/mi.2010.68 20944558

[75] Wang AJ Smith A Li Y Urban JF Jr Ramalingam TR Wynn TA. Genetic Deletion of IL-25 (IL-17e) Confers Resistance to Dextran Sulfate Sodium-Induced Colitis in Mice. Cell Biosci (2014) 4:72. 10.1186/2045-3701-4-72 25937893 PMC4417544

[76] Trembleau S Penna G Bosi E Mortara A Gately MK Adorini L. Interleukin 12 Administration Induces T Helper Type 1 Cells and Accelerates Autoimmune Diabetes in NOD Mice. J Exp Med (1995) 181(2):817–21. 10.1084/jem.181.2.817 PMC2191867 7836934

[77] Shao S He F Yang Y Yuan G Zhang M Yu X. Th17 Cells in Type 1 Diabetes. Cell Immunol (2012) 280(1):16–21. 10.1016/j.cellimm.2012.11.001 23246831

[78] Emamaullee JA Davis J Merani S Toso C Elliott JF Thiesen A. Inhibition of Th17 Cells Regulates Autoimmune Diabetes in NOD Mice. Diabetes (2009) 58(6):1302–11. 10.2337/db08-1113 PMC2682686 19289457

[79] Kumar P Natarajan K Shanmugam N. High Glucose Driven Expression of Pro-Inflammatory Cytokine and Chemokine Genes in Lymphocytes: Molecular Mechanisms of IL-17 Family Gene Expression. Cell Signal (2014) 26(3):528–39. 10.1016/j.cellsig.2013.11.031 24308966

[80] Su X Huang Q Chen J Wang M Pan H Wang R. Calycosin Suppresses Expression of Pro-Inflammatory Cytokines Via the Activation of P62/Nrf2-Linked Heme Oxygenase 1 in Rheumatoid Arthritis Synovial Fibroblasts. Pharmacol Res (2016) 113(Pt A):695–704. 10.1016/j.phrs.2016.09.031 27678042

[81] Lavocat F Ndongo-Thiam N Miossec P. Interleukin-25 Produced by Synoviocytes has Anti-inflammatory Effects by Acting as a Receptor Antagonist for Interleukin-17A Function. Front Immunol (2017) 8:647. 10.3389/fimmu.2017.00647 28620392 PMC5449741

[82] Min HK Won JY Kim BM Lee KA Lee SJ Lee SH. Interleukin (IL)-25 Suppresses IL-22-Induced Osteoclastogenesis in Rheumatoid Arthritis Via STAT3 and P38 Mapk/IkappaBalpha Pathway. Arthritis Res Ther (2020) 22(1):222. 10.1186/s13075-020-02315-8 32972460 PMC7517649

[83] Kaiwen W Zhaoliang S Yinxia Z Siamak SS Zhijun J Yuan X. Changes and Significance of IL-25 in Chicken Collagen II-induced Experimental Arthritis (Cia). Rheumatol Int (2012) 32(8):2331–8. 10.1007/s00296-011-1955-2 21626028

[84] Goldenberg MM. Multiple Sclerosis Review. P T (2012) 37(3):175–84. PMC3351877 22605909

[85] Turner DA Haile Y Giuliani F. IL-25 Prevents T Cell-Mediated Neurotoxicity by Decreasing Lfa-1 Expression. J Neuroimmunol (2013) 265(1-2):11–9. 10.1016/j.jneuroim.2013.10.006 24196277

[86] Sonobe Y Takeuchi H Kataoka K Li H Jin S Mimuro M. Interleukin-25 Expressed by Brain Capillary Endothelial Cells Maintains Blood-Brain Barrier Function in a Protein Kinase Cepsilon-dependent Manner. J Biol Chem (2009) 284(46):31834–42. 10.1074/jbc.M109.025940 PMC2797254 19776017

[87] Ma K Li J Wang X Lin X Du W Yang X. Tlr4(+)Cxcr4(+) Plasma Cells Drive Nephritis Development in Systemic Lupus Erythematosus. Ann Rheum Dis (2018) 77(10):1498–506. 10.1136/annrheumdis-2018-213615 29925508

[88] Tsokos GC. Systemic Lupus Erythematosus. N Engl J Med (2011) 365(22):2110–21. 10.1056/NEJMra1100359 22129255

[89] Selvaraja M Abdullah M Arip M Chin VK Shah A Amin Nordin S. Elevated interleukin-25 and Its Association to Th2 Cytokines in Systemic Lupus Erythematosus With Lupus Nephritis. PloS One (2019) 14(11):e0224707. 10.1371/journal.pone.0224707 31697750 PMC6837487

[90] Guo C Zhou M Zhao S Huang Y Wang S Fu R. Innate Lymphoid Cell Disturbance With Increase in ILC1 in Systemic Lupus Erythematosus. Clin Immunol (2019) 202:49–58. 10.1016/j.clim.2019.03.008 30926441 PMC8191378

[91] Ma K Wang X Shi X Lin X Xiao F Ma X. The Expanding Functional Diversity of Plasma Cells in Immunity and Inflammation. Cell Mol Immunol (2020) 17(4):421–2. 10.1038/s41423-019-0308-z PMC7109103 31649308

[92] Fox RI. Sjogren’s Syndrome. Lancet (2005) 366(9482):321–31. 10.1016/S0140-6736(05)66990-5 16039337

[93] Zhao X Gan Y Jin Y He J Jia R Li Y. Interleukin 17E Associates With Haematologic Involvement and Autoantibody Production in Primary Sjogren’s Syndrome. Clin Exp Rheumatol (2021) 39(2):378–84. 32573420

[94] Su J Xie C Fan Y Cheng W Hu Y Huang Q. Interleukin-25 Enhances the Capacity of Mesenchymal Stem Cells to Induce Intestinal Epithelial Cell Regeneration. Am J Transl Res (2017) 9(12):5320–31. PMC5752884 29312486

